# Supplementary material for: The causal role of circulating inflammatory markers in osteoporosis: a bidirectional Mendelian randomized study
Source: Front Immunol. 2024 Jul 18;15:1412298. doi: 10.3389/fimmu.2024.1412298 (PMC11291241; doi:10.3389/fimmu.2024.1412298)
Supplement: Supplementary file 1 [file DataSheet_1.zip › supplementary material/OSTEOPOROSIS-CIM/GCST90274797.tsv.gz_forest.pdf]

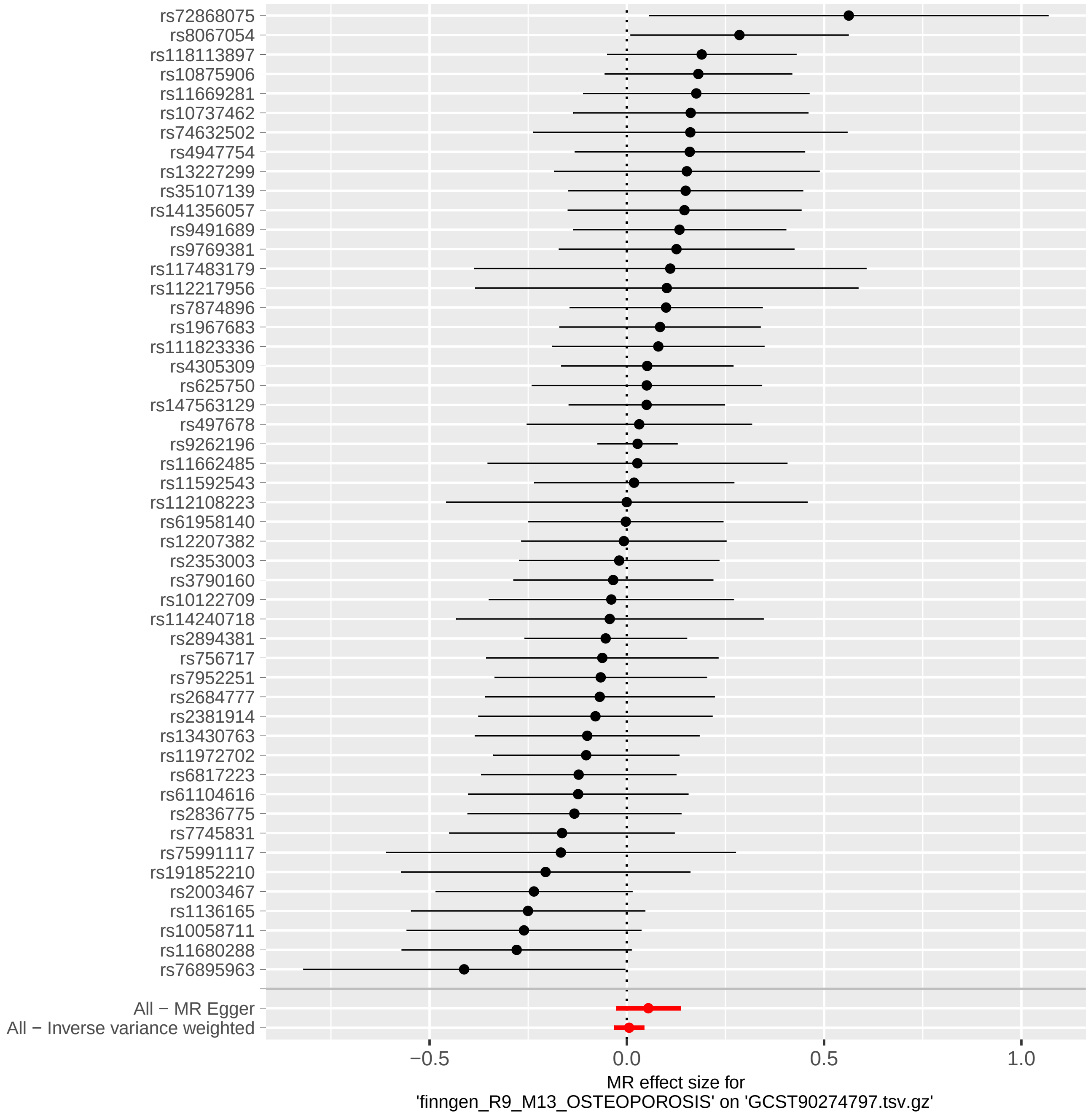
rs72868075
rs8067054
rs118113897
rs10875906
rs11669281
rs10737462
rs74632502
rs4947754
rs13227299
rs35107139
rs141356057
rs9491689
rs9769381
rs117483179
rs112217956
rs7874896
rs1967683
rs111823336
rs4305309
rs625750
rs147563129
rs497678
rs9262196
rs11662485
rs11592543
rs112108223
rs61958140
rs12207382
rs2353003
rs3790160
rs10122709
rs114240718
rs2894381
rs756717
rs7952251
rs2684777
rs2381914
rs13430763
rs11972702
rs6817223
rs61104616
rs2836775
rs7745831
rs75991117
rs191852210
rs2003467
rs1136165
rs10058711
rs11680288
rs76895963
All − MR Egger
All − Inverse variance weighted
−0.5
0.0
0.5
1.0
MR effect size for
'finngen_R9_M13_OSTEOPOROSIS' on 'GCST90274797.tsv.gz'
